# Benefits of Parent Training in the Rehabilitation of Deaf or Hard of Hearing Children of Hearing Parents: A Systematic Review

**DOI:** 10.3390/audiolres11040060

**Published:** 2021-12-13

**Authors:** Ilaria Giallini, Maria Nicastri, Laura Mariani, Rosaria Turchetta, Giovanni Ruoppolo, Marco de Vincentiis, Corrado De Vito, Antonio Sciurti, Valentina Baccolini, Patrizia Mancini

**Affiliations:** 1 Department of Sense Organs, University Sapienza of Rome, 00185 Rome, Italy; ilaria.giallini@uniroma1.it (I.G.); maria.nicastri@uniroma1.it (M.N.); laura.mariani@uniroma1.it (L.M.); rosaria.turchetta@uniroma1.it (R.T.); giovanni.ruoppolo@uniroma1.it (G.R.); marco.devincentiis@uniroma1.it (M.d.V.); 2 Department of Public Health and Infectious Diseases, University Sapienza of Rome, 00185 Rome, Italy; corrado.devito@uniroma1.it (C.D.V.); antonio.sciurti@uniroma1.it (A.S.); valentina.baccolini@uniroma1.it (V.B.)

**Keywords:** parent training, cochlear implant, hearing aid, deaf, hard of hearing, children

## Abstract

The present study is a systematic review on the effectiveness of Parent Training (PT) and coaching in deaf and hard of hearing (DHH) rehabilitation programs which reviews and synthesizes the existing body of evidence to assess the benefits of these programs in enhancing parents’ sensitivity, responsivity and promoting language development in DHH children during the first years after HA fitting or CI activation. Five published studies met the Population, Intervention, Comparison and Outcomes (PICO) inclusion criteria and were eligible to be included, but heterogeneity in terms of the study design, interventions and outcomes did not allow for performing a meta-analysis. All included studies shared the view that a parent’s learning is a circular (rather than frontal) process, and the results appear promising in terms of enhancing parents’ responsiveness and promoting DHH child language development. Nevertheless, the available evidence was judged to not be robust enough due to limitations in the studies’ designs. Further high-quality evidence is needed to evaluate the true degree of clinical value and the cost effectiveness of PT programs aimed at increasing parents’ responsiveness to their DHH children.

## 1. Introduction

In cases of congenital sensorineural hearing loss, early and timely intervention is considered the “gold standard” treatment to be initiated during the period of maximum neural plasticity in order to reduce the sensory deficits in the auditory system and promote cortical maturation [1]. Notably, a considerable number of deaf or hard of hearing (DHH) children who receive early hearing aid (HA) amplification or cochlear implantation (CI) achieve normal cognitive levels and good language skills, with developmental trajectories similar to their hearing peers [2]. However, a great deal of variability in the results for auditory, linguistic and academic outcomes still exists [1]. This variability depends on a complex set of interactions, including age at onset of hearing loss, degree of hearing impairment, degree of sensory deprivation, adequacy of hearing intervention and the type and quality of rehabilitation programs as well as social, parental and psychological factors [3].

Timely intervention with the most appropriate fitted hearing technology may exploit elevated early neural plasticity and may prevent or reduce the risk of dysfunction in brain maturation, increasing the probability of an early and rich auditory and linguistic experience. Stimulation of the auditory system during periods of maximal receptiveness (sensitive periods) is essential for its normal development [3]. Additionally, brain plasticity at the age of HA amplification or CI is a factor significantly affecting neurodevelopmental outcomes; complex auditory functions and speech perception cannot be comprehensively established when hearing restoration is performed at an old age, since some aberrant developmental steps in the synaptic counts, plasticity and network properties have taken place during the period without hearing [3].

Nevertheless, early and timely auditory restoration is not the only factor that is needed for DHH children to achieve successful hearing outcomes. The involvement of parents in a clear, full and concrete way in the intervention and rehabilitation program plays a very relevant and important role. During the last decade, the ability of parents to establish responsive interactions in daily activities and to use adequate linguistic input has been considered more and more crucial [4,5,6]. Parents’ responsiveness and sensibility play a critical role in promoting language, socio-emotional and cognitive development in both hearing children (HC) [7] and DHH children [8].

Caregivers, in fact, undertake a fundamental role in encouraging the infant’s involvement in communicative exchanges, empathically identifying themselves with a child’s moods, motivations and emotional states. Rhythmic and prosodic variations of the voice constitute privileged channels for the transmission of emotions, and it is precisely these passages of emotional expressions from mother to newborn and from newborn to mother that defines a “close mental contact” between partners [9]. On the other hand, the emotional disturbance of one of the partners, such as in cases of maternal depression, can impede the possibility of a successful intersubjective experience, with possible negative consequences for the psychological growth of the child [10].

Sensitive and responsive parenting may support and scaffold the child to achieve the internal regulation necessary to support more complex social, emotional and cognitive development [7,11]. For example, during early interactions in their first year of life, joint attention (i.e., the ability to share attention and interest toward external objects or events) is strongly influenced by maternal scaffolding [12,13]. Between 9 and 12 months, infants develop a basic ability that allows them to use shared linguistic symbols: the ability to think of the other as “intentional agents” and to share with them mental states. During the second year of life, this “mind sharing” ability develops more and more mature communication skills, such as the use of gestures of indication with requesting and declarative forms. This denotes not only the presence in the child of the concept of agency but also the awareness of others’ different mental states that can be modified through interpersonal communication [14]. It is therefore clear that the development of language begins before the possibility of using words and sentences, and it rests significantly on the quality of social interactions, which can support or hinder early prelinguistic communicative intents. Oral development necessarily requires early exposure to language and talking to a child is part of a communicative and relational framework made up of primarily emotional exchanges.

In the cases of hearing parents of DHH children, a number of factors may alter this natural parental responsiveness, such as the effect of limitations on the child’s access to sounds [15], the modification of parental interactions with their DHH children [16,17] and the psychological status of the parents [18,19]. In cases of congenital deafness in children, if caregivers perceive themselves as incapable or inadequate or they lack sufficient psychological or social resources, their communication might be characterized by reduced tuning, poor sensitivity and low contingency. In the event that these relational elements become embedded, they will strongly interfere with the development of a warm, welcoming, responsive and functional context for the child. Thus, feelings of “communication incompetence” can lead parents to a reduction in and exhaustion of conversational and interactive exchanges with their DHH child [20], often accompanied by an increase in intrusive, directive and less flexible behaviors [15,21]. Furthermore, reduced parental sensitivity and responsivity is associated with a disturbance in the executive functions (inhibitory control) and at-risk language outcomes in young DHH children. Conversely, this inhibitory control, when scaffolded by positive parental behaviors, may be critically important for robust language development in children who are DHH [22].

In recent decades, there has been an increasing promotion of family-centered programs for DHH children. A growing number of studies have highlighted that variable such as parental active involvement in therapy and parental communicative styles affect the long-term outcomes in DHH children [8,15,20,23,24]. Moreover, higher levels of parental distress have been associated with poorer communication, social and emotional development and higher rates of behavioral problems in DHH children [25].

The importance of putting families first in the intervention and rehabilitation of hearing loss in infancy was formally recognized when an international conference took place in Bad Ischl (Austria) in June 2012 with the aim of reaching a consensus on the basic principles to be adopted in Family-Centered Early Intervention (FCEI) with DHH children [8].

At the end of the conference, a document with a series of 10 foundational principles was produced with the goal of promoting a widespread, internationally agreed upon implementation of evidence-based interventions for FCEI with DHH children and their families. Four of these principles explicitly state the need for a family and provider partnership (Principle 2), informed choices (Principle 3), family social and emotional support (Principle 4) and optimization of family–infant interaction (Principle 5) [8].

The centrality of the family in the rehabilitation pathway of a DHH child is the focus of Auditory Verbal Therapy (AVT). This is a rehabilitation approach that aims to promote speech, linguistic, communicative and cognitive skills through listening as the main sensory modality, actively involving parents as the primary agents for change and development in their children [26]. Parents are an important component in each AVT session, and habilitation goals are planned soon after diagnosis according to individual needs and possibilities [26]. During AVT sessions, parents learn about their child’s current stage of development and define goals with the AVT practitioner by referencing the following developmental steps: learning and practice strategies to enhance listening, speech, communication, language and cognition in everyday interactions, creating adequate listening environments and home-enriched experiences [26].

Studies on the efficacy of an AVT parent-centered approach are increasing, and a recent systematic review [27] concluded that retrospective and longitudinal studies revealed statistically significant improvements in both expressive language and auditory comprehension for children involved in AVT programs compared with other traditional approaches, such as total communication, bilingual-bicultural or auditory-oral methods. Limitations in the quality of the published studies, however, make the results difficult to generalize. None of the published studies had an experimental design with participants’ randomization, and most of them did not report on the statistical power of their results. Potential cost–benefit advantages were not mentioned nor was the level of evidence rated from IIa to IIb [27].

Another strand of research focused its interest on developing specific programs of parent training (PT) for DHH children starting from the experiences gained in other domains of communication disorders, such as language delay [28,29] or autism spectrum disorders (ASDs) [30]. The goal of these PT programs was the enhancement of parental responsiveness and interaction-communication skills while providing a more supportive environment for communicative development in children [29]. Studies on the effectiveness of these PT programs showed a positive effect from the training both in parents’ sensitivity and responsivity and children’s communication outcomes in subjects affected by language delay [28,29] and ASDs [30]. Moreover, an indirect effect was observed in the increment to parents’ sensitivity and responsiveness, as it facilitated the children’s social, emotional and cognitive competence [7]. The effects of the adoption of these PT programs in the (re)habilitation of DHH children using HA or CI solutions are promising in that they might help parents to change their style of interaction with their DHH children, improving their confidence in adopting more attuned and responsive ways of communicating. A recent scoping review examined how parental training and coaching is conceptualized and implemented in DHH listening and spoken language practice. In describing the impact of caregiver coaching, it suggested that families of children who are DHH can effectively learn to implement specialized communicative strategies through coaching [31].

To date, however, no systematic review has evaluated the effectiveness of parent training and coaching in DHH rehabilitation programs. A systematic review could provide the gold standard evidence base to build future trials based on feasibility and effectiveness and to identify more effective ways of educating parents of DHH children with HA or CI. The primary objective of this study, therefore, was to review and synthesize the existing body of evidence to assess the benefits of PT programs in enhancing parents’ sensitivity and responsivity and promoting the language development of DHH children during the first years after HA fitting or CI activation.

## 2. Materials and Methods

The methods are reported according to the Preferred Reporting Items for Systematic Reviews and Meta-Analyses (PRISMA) checklist [32].

### 2.1. Eligibility Criteria

The PICO framework was followed to define the criteria for inclusion of participants, intervention, comparators, outcomes and study designs.

The participants included DHH children with hearing loss ranging from mild to profound using HA or CI and aged 0–6 years, including their parents. Studies on school-aged children were not included.

The intervention or object of investigation was the participation of one or both parents in a PT plan with the aim of increasing parents’ attunement, sensitivity and responsiveness during parent–child interactions and enrich the listening and linguistic environment around the child. Comparators were represented by parents who did not receive PT and their DHH children who attended listening, speech and language therapy.

Furthermore, the study had to apply a set of defined clinical outcomes regarding changes in the quantity or quality of interactions between parents and their children and the communication development of the children. The primary outcomes included measures of parent child-interaction (e.g., video analysis, observation grids or checklists such as Cole’s checklist or LENA), measurements of child communicative skills (e.g., McArthur questionnaire, standardized tests for speech and lexical or morpho-syntactic competences) and adverse effects, if any (e.g., PT abandonment).

There were no restrictions regarding the duration of a follow-up.

The following study designs were included: retrospective or prospective studies, randomized controlled trials, non-randomized controlled trials, before-and-after studies with active and control groups and before-and-after studies without control groups. Articles reporting expert opinions, practice guidelines, case reports, case series, conference abstracts and book chapters were excluded.

### 2.2. Search Strategy

The literature search was conducted on 7 July 2021. The following databases were searched: PubMed, CINAHL (via EBSCO host), Cochrane Library, Scopus, Citations Indexes of Web of Science, ISRCTN Registry and ClinicalTrials.Gov (Home—ClinicalTrials.gov; accessed on 18 September 2021). All databases were completed with no time, language, document type or publication status limitations. The search terms were collected based on free text and controlled vocabularies (Medical Subject Headings and CINHAIL headings) and were developed in relation to the population and intervention of interest: (“child” OR “infant”) AND (“hearing aid” OR “cochlear implant” OR “deaf” OR “hearing impair” OR “hearing loss” OR “hard of hearing” OR “D/HH” OR “DHH”) AND (“course” OR “parent training” OR “training course” OR “parent coaching” OR “parent implemented treatment”). The strings were adjusted for each database while maintaining a common overall architecture (Table S1).

Additional information was identified manually through snowballing of the reference lists from included studies, as well as screening of related articles by shortlisted authors to identify any relevant articles that may not have been returned by the initial database searches.

### 2.3. Study Selection

Before starting the review, the selection strategies were shared by the two reviewers (G.I. and N.M.) to ensure consistency of the data collection process. Two investigators (G.I. and N.M.) independently screened all identified references to decide eligibility according to the PICO criteria by reading the title or abstract. The full text was obtained for articles which appeared to satisfy the eligibility criteria or for those where there was some uncertainty (i.e., insufficient information to make a clear decision). We did not need to contact the study authors for further information to resolve eligibility issues. Discrepancies were resolved through discussion between the investigators.

### 2.4. Data Collection and Synthesis

Data collection was conducted by G.I. and N.M. independently but in duplicate for every included record. Specifically, a standardized data abstraction form was used to retrieve information on the study details (e.g., sponsorship source, country and setting), first author’s contact details (name, institution, email and postal address), study design, population (inclusion or exclusion criteria and baseline characteristics), comparators, if present (inclusion or exclusion criteria and baseline characteristics), intervention(s) and follow-up duration, outcomes and main findings.

Given the low comparability of the studies in terms of study design, intervention and outcomes, it was not possible to perform a meta-analysis. Hence, a narrative synthesis of the results was carried out.

### 2.5. Quality Assessment

G.I. and N.M. independently assessed the risk of bias in the identified randomized studies through the revised Cochrane risk-of-bias tool for randomized trials-RoB2 [33], which rates the studies as “high risk”, “low risk” or “some concerns” in the following five domains: randomization process (D1), deviation from the intended interventions (D2), missing outcome data (D3), measurement of the outcome (D4) and selection of the reported result (D5).

For the non-randomized studies that were included, quality was assessed through the Downs and Black (1998) checklist [34]. This last checklist contains 27 items divided between five subscales: reporting (10 items), which assessed whether the information provided in the paper was sufficient to allow a reader to make an unbiased assessment of the findings of the study; external validity (3 items), which addressed the generalization of the study’s findings to the whole population from which the studied subjects were derived; bias (6 items), which addressed bias in the measurement of the intervention and the outcome; confounding (6 items), which addressed bias in the selection of the study subjects; and power (1 item), which assessed whether the negative findings from a study could be due to chance. All answers were scored 0 (“no” or “unable to determine”) or 1 (“yes”), with the exception of criterion 5, (“Are the distributions of principal confounders in each group of subjects to be compared clearly described?”) which was scored 0 (“no”), 1 (“partially”) or 2 (“yes”). The total maximum score was 28, with the study quality rated as excellent (26–28), good (20–25), fair (15–19) or poor (≤14) [35].

## 3. Results

**Figure 1:**
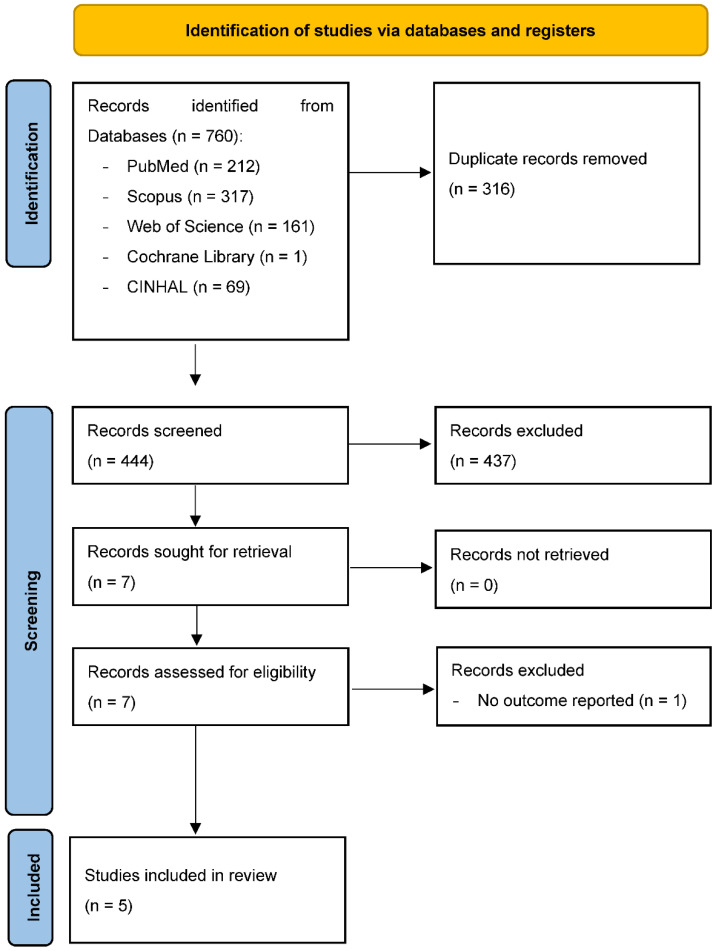
Selection of studies for the systematic review based on Preferred Reporting Items for Systematic Reviews and Meta-Analysis flow diagram (PRISMA 2020).

A total of 760 records were identified for screening. Following the removal of 316 duplicate publications, 444 records were subjected to a three-stage screening process (Figure 1).

The full texts of seven articles that passed the initial screening based on the title and abstract of the study were retrieved. Two articles did not meet the inclusion criteria; one did not contain adequate descriptions of the studied sample, and the assessment was performed through a subjective rating scale by a non-clinician rater [36], while the other was a case series study [37]. Five studies were included in the review process, and Table 1 summarizes their characteristics. Changes in parents’ responsiveness or sensitivity and in their CI children’s communicative and linguistic skills were evaluated. The comparators included parents who did not receive PT and their DHH children. One study was a pilot parallel randomized clinical trial [38], one was a before and after study without a control group [39], and the remaining three studies had a quasi-experimental design with an active group that received a “community-based early intervention plus PT” and a control group that received only “community-based early intervention” [40,41,42]. All the studies assessed the outcomes at different follow-up durations from 3 months [40] to 3 years [42].

### 3.1. Parental Responsivity and Sensitivity: Program Descriptions

All five included studies used PT with the aim of promoting parents’ sensitivity and responsiveness toward their DHH children during everyday activities and routines, facilitating communication and increasing, at the same time, the richness of the linguistic environment.

Glanemann et al. [40] developed the Muenster Parental Programme (MPP), consisting of six group sessions and two single sessions in weekly intervals flanked by one preparatory and one closing counseling session for each family, plus an additional refresher single training session when the child was between 24 and 30 months, giving a total of 11 sessions. The MPP is characterized by four core elements, represented by the creation of joint attention, the consistent and immediate reaction to child communicative attempts, the establishment of turn-taking and the use of expanded responses to the child. Parents are highly involved in each session and actively participate. Information is delivered through short lectures and video demonstrations. Video feedback is a core component of the single training session to allow parents to review their interaction with their children and reinforce parents’ awareness and confidence. After every session, the parents receive some homework for practice purposes and a parental handout.

Suskind et al. [41] applied a behavioral educational intervention named the ASPIRE curriculum, born within a project held by the Thirty Million Words program, with the aim of improving the language environment that low socio-economic status (SES) parents provide to their DHH children [43].

The ASPIRE curriculum is planned in 10 weekly hour-long modules, each based on the main research findings concerning language acquisition. They use easy to understand language and illustrate core concepts through means of animation and footage of caregivers and their children putting theory into practice. The interventions additionally employed behavioral change strategies using both video modeling of learned behaviors and the provision of quantitative linguistic feedback through Language Environment Analysis (LENA). LENA is a digital language recorder that returns the number of adult words a child is exposed to, the number of vocalizations the child attempts as well as a count of the conversational turns each adult and child engaged in over the course of a 16-h period.

Roberts [38] developed Parent-Implemented Communication Treatment (PICT) for DHH children. In PICT, parents are taught to encourage and reinforce the communication attempts of their children using four different strategies: visual (e.g., sitting face to face with the child, moving the object to the child’s attentional focus or waiting until the child looks before starting an interaction), interactive (e.g., following the child’s lead or choosing interesting toys), responsive (e.g., responding to all communicative attempts of the child or balancing turn taking) and linguistically stimulating (e.g., adding spoken words to the interaction or expanding child productions). Roberts [38] programmed a total of 26 h-long treatment sessions that took place once per week for 6 months. No video feedback was used, but during each session, the interventionist introduced a target strategy, modeled it with the child, let the parents practice the strategy with the child and, at the end, provided feedback and summarized the session for the parent.

Harrigan and Nikoloupolus [39] and Nicastri et al. [42] used the Hanen program “It Takes Two to Talk” (ITTT) [44,45]. The ITTT program was created by expert speech language pathologists at the Hanen Center of Toronto, and it is specifically designed for the parents of children with language delay. It is an evidence-based program that relies on extensive research [28,46,47], showing its effectiveness in changing the ways of parental interaction and improving children’s communication and language skills.

A core element in the ITTT approach is the consideration that the parents are the best language facilitators for their children. It is internationally acknowledged as a valuable method that helps parents reach a more responsive and less direct communicative approach in their daily interactions with their children. ITTT strategies [44,45] are believed to reduce parents’ directiveness as well as increase joint attention and shared focus between parents and their children. Additionally, they balance communicative turns and recognize and respond actively to child communicative attempts. They follow the child’s lead when adding language related to shared activities and linked to the child’s interests, mirroring the children and expanding their communication as well as using routines, play, book sharing and singing to create enriched linguistic environments around children.

Harrigan and Nikoloupulus [39] did not provide information regarding the total number of sessions and how many families participated in each group.

Nicastri et al. [42] specified that the organization of the PT program respected the ITTT manual indications, mixing group sessions for 3–5 families (total number of 9) with individual sessions (total number of 3). Group sessions where only parents were present were conducted according to the ITTT format, using teaching strategies indicated by the manual (e.g., problem solving, role play, brainstorming and video and sample discussions). Individual sessions were conducted with each parent and child pair. Here, the parents had the opportunity to practice the strategies and discuss them with a specialist. According to the ITTT model, video feedback was also used in individual sessions.

Harrigan and Nikolopoulus [39] did not specify which family components took part in PT. In the studies of Glanemann et al. [40] and Nicastri et al. [42], both mothers and fathers participated in PT, while in Suskind et al. [41] and in Roberts et al. [38], only mothers participated. Glanemann et al. [40] organized the training entirely in the clinic. Suskind et al. [41] and Roberts [38] organized training of the participants at home. Nicastri et al. [42] organized training partly in the clinic (group sessions) and partly at home (individual sessions). Harrigan and Nikolopoulus [39] gave information only on individual sessions at home, with nothing regarding the place of the group sessions.

### 3.2. Parental Responsivity and Sensitivity: Program Outcomes

All five studies reported significant changes in parents’ communicative behaviors after the training.

Harrigan and Nikolopoulus [39] involved 17 parents who all took part in PT. All participants were filmed at home during an interaction with their child before (within 2 months) and after (within 1 month) PT attendance, and video samples were then taken and transcribed orthographically.

With reference to Andersen-Wood and Smith’s (1997) principles and practice of pragmatics [48], caregivers’ interactive turns were counted and assigned to two behavior categories of “initiations” (parents initiate conversations or introduce new topics) and “responses” (parents respond to the child’s previous turn). No parent abandoned the training, and all of them expressed a high degree of satisfaction with the course. According to their study’s hypothesis, when comparing the pre-post count of parents’ behavior, they found a significant reduction in “initiatives” (median changed from 14 to 7, *p* = 0.01) and a significant increase in “responses” (median changed from 8 to 16, *p* = 0.0004). As a follow-up, video samples of 16 parents were taken 12 months after the end of PT, and the scores remained high for “responses” (16.5), but a slight increase was observed for “initiatives” (median changed from 7 to 8.5).

Glanemann et al. (2013) [40] involved 29 parents divided into two groups: the PT group (15 parents) and a control group (14 parents). The authors measured the parents’ behaviors, denoting responsiveness (dialogic echo and responsiveness to movement or action) and the duration of behavior signaling directiveness, defined as “initiative” (i.e., inadequate introduction of a new object or action or neglecting the child’s current focus of attention). These parental variables were set in relation to the child’s offers, recording three ratios (dialogic echo ratio, responsiveness to movement or action ratio and initiative ratio) that were computed through the Mangold Interact software tool [49]. The analysis was performed on a sample of 4 min, extracted in a standardized way from 15-min long video samples of parent and child communication recorded in the clinic. The coding of the relevant parental behavior was performed by a trained rater, who was blind to the condition and test time (pre-post). Inter-rater reliability was assessed by randomly selecting 10% of the data and having a second rater undertake the same assessment who was also blind to the condition and test time, with a Pearson’s correlation rating from 0.99 for the dialogic echo to 0.69 for the duration of initiative. Between the pre-post assessments, there were no dropouts from the PT group and only one drop out from the control group. The parents in the PT group showed a significant increase in behaviors denoting responsiveness (pre-post differences for dialogic echo showed t = −3.806, df = 14, *p* = 0.002; pre-post differences for responsiveness showed a t = −4.673, df = 14, *p* < 0.001) and a significant decrease in initiative behaviors (t = 5.687, df = 14, *p* < 0.001), denoting directiveness. No pre-post significant differences were detected in the control group.

Suskind et al. [41] studied 14 low SES mothers who were included in the treatment group and 17 low SES mothers in the control group. Five families in the PT group did not complete the baseline measures, and a further four families (two control and two treatment) dropped out mid-intervention.

The parents were assessed at baseline, 2 weeks before the beginning of PT, immediately after the end of PT (post 1), which lasted 2.5 months, and then 3 months later (post 2) for a total follow-up of 6 months. Two outcome measures were considered: video and LENA. For video outcomes, a 20-min free play session was recorded in the clinic using a standardized set of toys, and each mother was assessed interacting with her child. A trained research assistant blind to the condition transcribed the number of utterances, word types, word tokens and Mean Length of Utterances (MLU) for each video. For the LENA outcomes, the number of adult words (AWC) and conversational turns (CTC) were computed.

Furthermore, a 45-item survey with a 5-point Likert scale response was developed to assess parental knowledge of child language development, and it was undertaken with participants.

The mothers in the PT group significantly increased the number of words they used with their child (word tokens) and their MLU immediately after the training, although these differences did not persist at the 3-month follow-up. The authors concluded that ASPIRE intervention had a short-term positive impact on the language output of the caregivers, but it was impossible to draw conclusions regarding the long-term effects. A strong effect of treatment was instead found at both the post 1 and post 2 responses to the survey, denoting a stable increment in mothers’ knowledge about child language development.

In her randomized trial, Roberts [38] involved 9 mothers in the PT group and 11 mothers in the control group. One participant dropped out of the control group. The outcome data were collected at home at baseline (immediately before PT) and straight after the end of the treatment. The mothers as well as the assessors were aware of the experimental conditions. However, interobserver agreement was computed between the program assessors and two independent naïve raters, who scored 20% of all measures. The intraclass correlation coefficient was 0.80, indicating that the potential for experimenter bias was low. The assessors coded through the Mangold INTERACT software tool [49] the middle segment of a 10-min parent–child interaction at home and collected pre and post PT using a standard set of toys. The outcome was recorded as the percentage of adult utterances that included one of the communication strategies presented during PT. Differences in mothers’ use of treatment strategies between PT and the control group was large, with an effect size (ES) of 1.08 (*p* = 0.04).

Nicastri et al. [42] involved 14 mothers and 8 fathers both in PT and a control group. There were no dropouts during the study. The outcomes were collected within 1 month of PT’s beginning and within 1 month of the end of PT. The primary outcome was the score recorded with the Communication-Promoting Behaviors Checklist for Caregivers tool [50]. The checklist was completed by two trained speech therapists who were blind to the participant condition and who analyzed a video recording of parent–child interactions while following the Cole manual instructions [50]. The raters assessed each of 22 items and then calculated a mean score for all 22 items (overall score) as well as for each subcategory of the checklist (Sensitivity to Child, Response to Child, Shared Attention and General). Although a change was detected in the overall score as well as at each subcategory for both mothers and fathers in the PT and control groups, the parents from the PT group achieved higher scores than the control group parents (time for group interaction effects ranging from 74.5 to 160.1 (*p* < 0.001) for mothers and from 11.8 to 5.1 (*p* < 0.05) for fathers). The effect sizes exceeded 0.8. The secondary outcome was the score recorded in the Parent Stress Index Short Form questionnaire, but no significant differences were detected between the PT and control groups.

### 3.3. Child Communication Skills: Program Outcomes

Harrigan and Nikolopoulus [39] did not assess the participating children. All the other four studies [38,40,41,42] assessed the children of both the PT and control parents at baseline and at the end of the training. Suskind et al. [41] also collected data after a 3-month interval, and Nicastri et al. [42] also performed a longer follow-up, assessing them again 3 years after the end of PT.

Regarding the outcomes, Glanemann et al. (2013) [40] counted the total number of vocalizations of the 14 DHH children of the PT parents and of the 10 DHH children of the control parents, analyzing the video samples of interactions that were used for assessing parents’ communication behaviors. In the pre-PT assessments, there were no differences in the total number of vocalizations between the children of the PT and control groups (14.1 vs. 15.1, respectively, t = −226, df = 27, *p* = 0.823). However, the differences between the groups were significantly in favor of the children of the PT parents in the post PT assessment (16.5 vs. 9.6, respectively, t = 2.994, df = 27, *p* = 0.006).

Similarly, a greater improvement in the children of the PT parents was detected by Roberts [38] and Nicastri et al. [42]. Roberts [38], using the Communication and Symbolic Behavior Scale Behavior Sample [51], found a significant increase in the speech pre-linguistic subscale with a large effect size of 1.09 but none for the social or symbolic pre-linguistic skills.

Nicastri et al. [42] assessed the linguistic competencies of DHH children through the MacArthur–Bates Communicative Development Inventory (MCDI) [52,53] Gestures and Words Form immediately after the end of the training. Then, after 3 years, a set of Italian standardized tests for lexical and morpho-syntactic domains were utilized. The children of the PT group achieved better scores than the children of the control group for all the outcome measures both immediately after PT (significant time × group interaction effects F1,26 ranging from 5.4 to 13.5, *p* values < 0.05) and 3 years later (*p* values ranging from 0.019 to 0.007). The effect of PT was high at the end of the programs, and still, a large effect size was recorded at 3 years for the morpho-syntactic skills (ES 0.93). In the longer-term follow-up, the impact was instead smaller for lexical abilities (ES 0.34 and 0.31 for lexical production and comprehension, respectively).

Nicastri et al. [42] searched for a correlation between the improvement in parents’ responsivity and the increase in children’s communication skills at the end of PT, and they reported a positive relationship between the children’s word production and the mothers’ scores on the General subscale of Communication-Promoting Behaviors Checklist for Caregivers [50]. A greater effect of intervention on the CI children’s word production was observed when both parents took part in PT.

Suskind et al. [41] used the count of the number of children’s utterances, word types, word tokens and MLU, obtained by two blind transcribers that analyzed the 20-min video-recorded free play session, which were also utilized for the parents’ assessment. None of the measures significantly differed in the pre-post assessments, indicating no effects of PT in the children. The authors stated that “… regressions reveal only the normal increases in language capacity associated with growing older, but no effects associated with the ASPIRE intervention. The large standard errors in the child-level regressions suggest that children may receive some benefit from the intervention, but it is impossible to tell given the small sample size in the current experiment.” In detail, their analysis was performed respectively on 6/12 children at follow-up 1 and 7/12 children at follow-up 2.

### 3.4. Quality of the Included Studies and Potential Bias

The quality of the “before and after” study [39] and of the three quasi-experimental studies [40,41,42], assessed using the Downs and Black checklist (1998) [34], ranged from poor to fair (Table 1).

Harrigan and Nikolopoulus [39] did not comply to all the Downs and Black checklist domains, as they did not provide the information required to characterize the studied group of parents, nor did they include a control group, and the description of PT intervention was partial.

In the three quasi-experimental studies [40,41,42], sufficient information was provided to allow the reader to make an unbiased assessment of the studies’ findings. A representative sample of DHH children and their caregivers was recruited to foster the generalization of their findings to the whole population from which the studied subjects were derived (reporting and external validity). Instead, the risk of bias was more related to their internal validity and their power. In detail, they lacked participant randomization, adjustments for confounding in the statistical analysis and a statistical calculation of the size of the smallest intervention group sufficient to detect clinically relevant effects, with a chance value of less than 5%. Furthermore, all three studies were not blinded regarding the participants, who were not naïve to their experimental condition (risk of performance bias). Suskind et al. [41] and Glanemannn et al. [40] had nine and one participants that dropped out of the study, respectively, but they had not been considered in the statistical analysis nor appropriately described (risk of attrition bias).

The study by Roberts [38], assessed through the RoB2 checklist, had a low risk of bias regarding the randomization process (D1) and the selection of the reported results (D5). Some concerns arose from the effect of adhering to the interventions (D2), missing outcome data (D3) and the measurement of the outcomes (D4). In particular, the participants and assessors were not naïve to the experimental conditions (risk of performance and detection bias), and one participant dropped out from the control group without his data being considered in the analysis (risk of attrition bias).

## 4. Discussion

Parent training is a set of interventions providing caregivers with educational, psychological and rehabilitative processes, taught in order to help them to actively acquire knowledge, skills and strategies through mechanisms such as homework, modeling, or practicing skills [54].

This systematic review focused on existing research evaluating the effectiveness of PT programs in DHH rehabilitation from a twofold perspective: PT benefits on the level of sensitivity and the responsivity of HC parents toward their HA or CI children aged 0–6 years as well as PT benefits in the linguistic development of HA or CI children at the end of the training or at a long-term follow-up.

Five published studies met the PICO inclusion criteria and were eligible to be included.

All of them had in common the centrality of the family relational framework and the theoretical assumption that the development of language begins before the child has the opportunity or ability to use words [55,56,57]. The possibility of implementing the rehabilitation of a DHH child with an intervention focused on caregivers is based on the awareness that relational and environmental components play an important part in their communicative and linguistic outcomes [12,58,59,60]. Following this theoretical assumption, all the reported protocols have the common goal of promoting parental empowerment and responsiveness. Parental empowerment can be defined as the acquisition of a greater sense of mastery and awareness concerning their child’s language development, communicative limitations and needs linked to hearing loss.

Additionally, their understanding of the importance of communication support strategies is essential to achieve stronger language outcomes for their children. Parental responsiveness refers to the parents’ skills “to allow the child to be the leading person in the communication, to react immediately to its communicative attempts by imitating the child’s signals and responding to it in an expanding way” [40] and to the caregivers’ ability to attune to the child, to identify the child’s communicative attempts and to provide contingent responses [42]. Roberts [38] and Suskind et al. [41] did not explicitly use the term responsiveness in the primary objectives of their research. However, the precise description of the core elements of the two intervention programs contains explicit parental communication strategies aimed at engaging caregivers in increasing communication exchanges, language input and responsive communication behaviors.

The included studies also shared the view that parent learning is a circular (rather than frontal) process, consequently passing from the concept of “teaching” strategies to “guiding” parents to building their own knowledge, supporting their critical thinking about strategies and communicative behaviors to strengthen the child’s emerging listening and communicative skills.

To realize critical learning, active learning strategies were implemented such as the use of animations, real life footage of caregivers and their children, brainstorming or problem-solving activities. Additionally, presentation and direct practicing of specific listening or communication strategies, role playing, video analysis, video feedback or direct feedback on shared communication strategies were given by the interventionist to parents during parent–child interactions. This approach is the result of decades of research showing the advantages of active learning compared with passive learning approaches [54,61].

On these premises, all the protocols reported significant differences between the PT groups and control groups in all measures used to detect parental change as measured soon after the end of the PT programs. PT seems to show promise in increasing DHH parents’ empowerment and responsiveness. The authors used different assessment tools, but all concordantly found significant changes in parents’ behavior during interaction with their children, such as an increment in dialogic echo and imitation or repetition of the child’s movements or actions [40]. Additionally, there was an increased number of words and MLU used with the child [41], a growth in the visual, interactive, responsive and linguistically stimulating strategies used to reinforce the child’s communicative attempts [38,39] as well as an improvement in sensitivity, responsiveness, shared attention and generally positive behavior toward the child [42].

Simultaneously, three of the five studies found that the children of PT parents showed better scores in both prelinguistic communication [38,40] and speech or verbal skill [42] assessments. The advantage for the DHH children of PT parents was significant for subjects where both parents took part in PT, and these advantages were maintained at the 3-year follow-up [42].

The included studies highlight the potential for PT to promote learning and change in the parents of DHH children, driving them toward responses and behaviors that are more facilitative of their children’s communicative development. Parents should be considered as the primary agents of change engaged in the promotion of their children’s development, and this is particularly true for early communication and language development, where the quality and quantity of adult language input strongly influences language growth in both young HC and DHH children [4,5,7]. Within the general framework of family-centered practice [62], to train the parents of DHH children to use communicative and language facilitative strategies should be considered a key component in early intervention programs.

Nevertheless, several reflections arise from the present systematic review.

First, there is the paucity and heterogeneity of studies investigating the effectiveness of programs implemented for the families of DHH children. While the centrality of family in early intervention for DHH children is well established [8], and research is increasing on the effect of parents’ interaction and linguistic input on their children’s development [25,63,64,65,66], few studies have been published on the efficacy of training programs for the parents of DHH children in effectively enhancing parental responsiveness and interaction-communication skills. We identified only seven studies that investigated this domain, and between them, only five were eligible for the systematic review according to the PICO criteria. The eligible studies showed differences in the reference training models (e.g., PICT, ITTT, ASPIRE and MPP) and in specific procedures used to train the parents: exclusive, individual sesions [38,41] vs. mixed group and individual sessions [39,40,42]; service delivery models ranging from clinic-based [40] or solely home-based [38,41] to clinic combined with a home-based setting [42]; and caregivers who took part in the training (e.g., both parents [40,42] or only mothers [38,41]). The intensity of the interventions varied, as well as the follow-up durations and tools used to detect changes in the parents and the communicative development in the children. Additionally, the participants varied in SES across the studies; some were mixed lower and middle SES [42] while some were only low SES [41], and others did not specify this demographic information [38,39,40]. Heterogeneity makes generalization of the results difficult.

Secondly, the evidence of the studies was judged to range from poor to fair for the three quasi-experimental studies, and some concerns also arose from the clinical trial of Roberts [40]. The absence of a control group in Harrigan and Nikoloupolus [39] did not allow them to conclude as to whether changes detected in the PT parents were really imputable to the effect of the PT program or were rather due to the natural progressive communicative adjustments that parents make to adapt their way of interaction to the changing communication skills of their DHH children that evolve after HA prescription or CI, as well as their involvement in the listening and spoken language rehabilitation [67,68,69]. Furthermore, lack of randomization and sample size calculations were factors for potential bias in the three quasi-experimental studies. Randomization is the process through which all study participants have the same chance to receive all the available treatments, and as the sample size increases, it tends to produce groups that are comparable and match both their measured and unknown factors that may impact treatment response, thus reducing selection bias [70]. As confirmation of this, in Glanemann et al. [40], the PT and control groups were unbalanced regarding both parents (e.g., a different percentage of fathers and mothers between groups) and children (e.g., in bilateral-unilateral hearing loss ratio and in the degree of bilateral hearing between the groups). In addition, in the work of Suskind et al. [41], the children were unbalanced (significant differences in chronological age, degree of hearing loss and pre-PT video measures). Nicastri et al. [42] tried to match the PT and control groups both for parents and children but lacked sufficient explanation about the methodology followed to perform the matching. None of them calculated the sample size necessary to detect clinical meaningfulness between PT and control group interventions, with the consequent risk that the potential benefit of the treatment is discarded (type II error). Roberts [38], planning a randomized clinical trial, overcame these problems, but his study sample was very small (9 and 10 participants for the PT and control group, respectively), and he defined his study as a pilot experience. Estimates based on small pilot studies “… may often provide overly optimistic estimates of standard errors and effect sizes because they are typically conducted at single centeres with lower variability than would be found in a multicenter trial, with participants more representative of the ultimate target population” [70].

Furthermore, an issue common to all the above cited studies, typically found in research into the rehabilitation domains, is related to the blinding process [70]. Glanemann et al. [40], Suskind et al. [41] and Nicastri et al. [42] had blind assessors. Roberts’ assessors were naïve to the experimental conditions. None of the studies could make blind the participants nor the clinician who provided the training due to the difficulties associated with creating a true placebo in rehabilitation trials, and this therefore could be a cause of important performance bias [70]. For example, participants in the control group might seek other treatments, or researchers or clinicians might treat participants differently depending on which group they are in. This bias may inflate the estimated effect of the intervention. As a rule, when the subjects are not blinded, the treatment effects tend to be higher [71].

Three of the five included studies had some participants drop out [38,40,41], but they were not described nor considered in the statistical analysis using appropriate methods to estimate the effect of adhering to intervention. Participant attrition may alter the characteristics of the sample, and therefore, it is no longer representative of the original cohort, which might also unbalance the covariance of the variables so that the loss of participants with a particular attribute will affect the outcomes of a different but related dependent variable [72]. Therefore, this introduces a risk of bias and reduces the generalizability of the findings [72]. Glanemann [40] and Roberts [38] each had only one participant drop out, and both remained under 10% for participant attrition, considered the threshold under which the missed data does not introduce bias [34]. Suskind [41] instead ceded the data of seven (50%) and two (11.7%) subjects in the PT and control groups, respectively, despite all participants receiving a fee to take part in the study. The large quotient of participant attrition in the PT group could reflect on the acceptance, feasibility and adherence to a PT program in the particular social context of families with low SES that represented the study population of Suskind [41].

Finally, the designs of the included studies did not allow for the stability of the parents’ learning at the time. Glanemannn et al. [40], Nicastri et al. [42] and Roberts [38] assessed the quality of the parents’ interactions only at the end of the training, so it was not possible to know if the learned strategies were stable at a later time. Only Suskind et al. [41] included a parental follow-up 3 months after the end of PT and found that differences between the PT and control groups of the parents were not maintained, suggesting the possibility that parents can revert to a previous, less responsive communication modality with their DHH children.

## 5. Conclusions

Use of PT as a component part of the rehabilitation process after HA and CI appears promising for DHH children. PT seems to help parents become active learners and acquire a more responsive way of communicating with their DHH children, who in parallel appear to make significant progress in their preverbal and verbal skills. Nevertheless, the available evidence was judged to not be robust enough, ranging from poor to fair in quality and subject to bias due to limitations in the studies’ designs. Further high-quality evidence is needed to evaluate the true degree of clinical value and the cost effectiveness of PT programs aimed at increasing parents’ responsiveness to their DHH children. New studies could also help clinicians to understand which reference models could be more efficacious and to define the more effective combination of factors that could influence outcomes, from program intensity and duration of PT to the modality of performing the training (individual, group or mixed sessions), as well as the place where it should be implemented and the caregivers who should be involved.

## 6. Limitations

The present review was carried out using a standardized scientific methodology at every stage of the development process. Nevertheless, the small number of included studies and the lack of homogeneity in study design impeded the use of a quantitative approach to data analysis limiting the possibility to make definite statements or conclusions on the effectiveness of these kind of interventions.

Furthermore, the included studies were not addressing all the children and parents’ variables that, in a more extensive way, might have benefited from PT. Therefore, the outcome measures reported may not be fully indicative of PT’s effectiveness.

### Implications for Clinical Practice and Future Research

Despite the above-mentioned limitations, our study provides newsworthy insights for clinical practice and suggestions for future investigations. PT seems to have the potential to promote children’s communication and language development and could be integrated in rehabilitative clinical practice, but many questions remain open. Are there differences in outcomes following one reference training model rather than another? Which intensity of PT program could be considered more advantageous in terms of the cost-effectiveness ratio, considering outcomes, economic costs and energy and time investments for clinicians and parents? Which kind of delivery is more effective between the choices of home, clinic or mixed models? Could mothers be considered the preferential referents, or could the inclusion of both parents guarantee stronger benefits for child development, as seems to be the conclusion from the Nicastri et al. [42] study? Is there a risk of parents relapsing toward less responsive ways of communicating, and how do clinicians avoid a parent returning to more directive and less attuned forms of communication while interacting with their DHH children?

All these queries should be addressed in future research to define shareable and replicable protocols that could be effectively used in clinical practice.

## Figures and Tables

**Table 1:** Summary table of articles included in the review.

Reference	Study Design	Participants (PT-Trained)	Controls (No PT)	Participants Drop Out	Intervention	Assessments Measures	Outcomes	Follow-Up Duration	Quality of the Study
Harrigan and Nikoulopolus, 2002 [39] Great Britain	Before and after study	Parents N = 17 No other information	Absent	PT-trained = 0	ITTT	Video analysis of pre-post count of parents’ initiatives and responses	Significant increase in responses and decrease in initiatives, denoting an increment to parents’ responsiveness	No information	Downs and Black checklist: 11 (poor)
Glanemann et al., 2013 [40] Germany	Quasi-experimental prospective study	Parents N = 15 (14F, 1M) Mean age = 33.9 (sd 4.8) Children N = 14 (7F, 7M) Age = 6.8 months (sd 5.2) Heterogeneous degree of hearing loss and typology of hearing devices 4 children with moderate or severe developmental delay	Parents N = 14 (9F, 5M) Mean age = 32.7 (sd 5.6) Children Control N = 10 (6F, 4M) Age = 5.7 months (sd 2.0) Heterogeneous degree of hearing loss and typology of hearing devices 3 subjects with severe developmental delay	PT-trained = 0 No PT = 1	Muenster Parental Programme	Video analysis of pre-post parents dialogic echo and responsivity ratios for caregivers through Interact software Video analysis of children pre-post for total numbers of vocalizations	Significant differences between PT and control group in post-PT assessment both for parent and child measures	4 months	Downs and Black checklist: 16 (fair)
Suskind et al., 2016 [41] United States of America	Quasi-experimental prospective study	Parents N = 14 (14F) Mean age = 25.8 (sd 4.9) Children N = 14 (8F, 6M) Age = 17.0 months (sd 10.0) Heterogeneous degree of hearing loss and typology of hearing devices Family low-socioeconomic status	Parents N = 17 (17F) Mean age = 30.5 (sd 9.5) Children Control N = 17 (10F, 7M) Age = 27.8 months (sd 13.9) Heterogeneous degree of hearing loss and typology of hearing devices Family low-socioeconomic status	PT-trained = 7 No PT = 2	ASPIRE curriculum	45-item survey assessing parent knowledge of child language development Caregivers and children count utterances, word types, word tokens, Mean Length of Utterance (MLU) and conversational turns collected through video analysis and LENA used during caregiver–child interactions	Significant differences between PT and control group in post-PT assessment for parents’ measures but not for children Loss of significance in differences between PT and control group parents for word types, word token and MLU count at follow-up 2	6 months	Downs and Black checklist: 14 (poor)
Roberts, 2019 [38] United States of America	Parallel randomized controlled trial	Parents N = 9 (9F) Mean age not reported Children N = 9 (3F, 6M) Age = 10.1 months (sd 4.26) Heterogeneous degree of hearing loss. All children wore hearing aids	Parents N = 10 (9F) Mean age not reported Children N = 10 (4F, 6M) Age = 13.7 months (sd 7.77) Heterogeneous degree of hearing loss. All children wore hearing aids	PT-trained = 0 No PT = 1	Parent-Implemented Communication Treatment (PICT)	Percentage of parental use of communication strategies obtained by analyzing video recorded parent–child interaction samples through the Mangold INTERACT software (Mangold, 2015) Children’s prelinguistic communication skills assessed through the Communication and Symbolic Behavior Scale Behavior Sample (Wetherby and Prizant, 2003)	Significant differences in percentage of communication strategies used by parents in favor of PT parents Larger gain in speech prelinguistic skills in children of PT parents than in children of the control group	6 months	RoB2 risk of bias *: D1: low D2: some concerns D3: some concerns D4: some concerns D5: low
Nicastri et al., 2020 [42] Italy	Quasi-experimental prospective study	Parents N = 22 (14F, 8M) Mean age = 35.4 years (sd 5.4) for mothers and 40.4 years (sd 4.3) for fathers Children N = 14 (7F, 7M) Age = 25.6 months (sd 6.3) All children had severe-profound hearing loss and wore cochlear implants	Parents N = 22 (14F, 8M) Mean age = 37.8 years (sd 5.3) for mothers and 44.5 years (sd 4.9) for fathers Children N = 14 (7F, 7M) Age = 26.2 months (sd 6.2) All children had severe to profound hearing loss and wore cochlear implants	PT-trained = 0 No PT = 0	Hanen ITTT program	Parents’ quality of interaction based on a video-recorded sample analysis performed through the Communication-Promoting Behaviours Checklist for Caregivers (Cole, 1992) Children’s MacArthur–Bates Communicative Development Inventory Italian standardized language tests to assess child lexical and morphosyntactic skills	Significant differences in the scores at Communication-Promoting Behaviours Checklist for Caregivers in favor of PT parents at the end of PT Higher scores in all tests in children of PT parents compared to children in the control group at the end of PT and even 3 years after the end of PT	10.5 months caregivers 3 years children	Downs and Black checklist: 18 (fair)

* RoB2 domains are D1: risk of bias arising from randomization process; D2: risk of bias due to deviations from the intended interventions; D3: missing outcome data; D4: risk of bias in measurement of the outcome; and D5: risk of bias in selection of the reported result.

## Supplementary Materials

The following are available online at https://www.mdpi.com/article/10.3390/audiolres11040060/s1, Table S1. Search strategies used in the systematic review.
