# Supplementary material for: Benefits of Parent Training in the Rehabilitation of Deaf or Hard of Hearing Children of Hearing Parents: A Systematic Review
Source: Audiol Res. 2021 Dec 13;11(4):653–72. doi: 10.3390/audiolres11040060 (PMC8698273; doi:10.3390/audiolres11040060)
Supplement: Supplementary file 1 [file audiolres-11-00060-s001.zip › audiolres-1423918-supplementary.pdf]

Supplementary

Table S1. Search strategies used in the systematic review.

Database	Search string
PubMed	(“deaf*” OR “hearing impair*” OR “hearing loss” OR “hard of hearing” OR “D/HH” OR “DHH” OR "Persons With Hearing Impairments"[MeSH Terms] OR "Hearing Loss"[MeSH Terms]) AND (“child*” OR “infant*” OR "Child"[MeSH Terms] OR "Infant"[MeSH Terms]) AND (“hearing aid*” OR “cochlear implant*” OR "Hearing Aids"[MeSH Terms] OR "Cochlear Implants"[MeSH Terms]) AND (“course*” OR “parent training” OR “training course*” OR “parent coaching” OR “parent implemented treatment”)
Scopus	TITLE-ABS-KEY(“deaf*” OR “hearing impair*” OR “hearing loss” OR “hard of hearing” OR “D/HH”OR “DHH”) AND TITLE-ABS-KEY(“child*” OR “infant*”) AND TITLE-ABS-KEY(“cochlear implant*” OR “hearing aid*”) AND TITLE-ABS-KEY(“course*” OR “parent training” OR “training course*” OR “parent coaching” OR “parent implemented treatment”)
Web of Science	TS=(“deaf*” OR “hearing impair*” OR “hearing loss” OR “hard of hearing” OR “D/HH”OR “DHH”) AND TS=(“child*” OR “infant*”) AND TS=(“cochlear implant*” OR “hearing aid*”) AND TS=(“course*” OR “parent training” OR “training course*” OR “parent coaching” OR “parent implemented treatment”)
Cochrane Library	((“deaf*” OR “hearing impair*” OR “hearing loss” OR “hard of hearing” OR “DHH”):ti,ab,kw OR "Persons With Hearing Impairments"[MeSH Descriptor] OR "Hearing Loss"[MeSH Descriptor]) AND ((“child*” OR “infant*”):ti,ab,kw OR "Child"[MeSH Descriptor] OR "Infant"[MeSH Descriptor]) AND ((“cochlear implant*” OR “hearing aid*”):ti,ab,kw OR "Hearing Aids"[MeSH Descriptor] OR "Cochlear Implants"[MeSH Descriptor]) AND (“course*” OR “parent training” OR “training course*” OR “parent coaching” OR “parent implemented treatment”):ti,ab,kw
CINHAL Database	(“deaf*” OR “hearing impair*” OR “hearing loss” OR “hard of hearing” OR “D/HH” OR “DHH” OR (MM "Hearing Loss, Partial+") OR (MM "Deafness+")) AND (“child*” OR “infant*” OR (MM "Child+") OR (MM "Infant+")) AND (“hearing aid*” OR “cochlear implant*” OR (MM "Hearing Aids+") OR (MM "Cochlear Implant+")) AND (“course*” OR “parent training” OR “training course*” OR “parent coaching” OR “parent implemented treatment”)
ISRCTN Registry	(“deaf*” OR “hearing impair*” OR “hearing loss” OR “hard of hearing” OR “D/HH” OR “DHH”) AND (“child*” OR “infant*”) AND (“hearing aid*” OR “cochlear implant*”) AND (“course*” OR “parent training” OR “training course*” OR “parent coaching” OR “parent implemented treatment”)
ClinicalTrials.gov	(“deaf*” OR “hearing impair*” OR “hearing loss” OR “hard of hearing” OR “D/HH” OR “DHH”) AND (“child*” OR “infant*”) AND (“hearing aid*” OR “cochlear implant*”) AND (“course*” OR “parent training” OR “training course*” OR “parent coaching” OR “parent implemented treatment”)
